# First Italian Experience with the Oxinium Metal-Backed Fixed-Bearing Medial Journey II Unicompartmental Knee System: Promising Short-Term Outcomes of 145 Cases

**DOI:** 10.3390/jcm13051303

**Published:** 2024-02-25

**Authors:** Federico D’Amario, Luca De Berardinis, Giacomo Zanon, Fjorela Qordja, Umberto Vitale, Antonio Pompilio Gigante

**Affiliations:** 1 Orthopedic Unit, Humanitas San Pio X, Via Francesco Nava, 31, 20159 Milano, Italy; federico.damario@gmail.com (F.D.); zanon.g@libero.it (G.Z.); 2 Clinical Orthopedics, Department of Clinical and Molecular Sciences, School of Medicine, Università Politecnica delle Marche, Via Tronto, 10/a, 60126 Ancona, Italy; qordja.fjorela@gmail.com (F.Q.); a.p.gigante@staff.univpm.it (A.P.G.); 3 IRCCS Humanitas Research Hospital, Via Manzoni, 56, Rozzano, 20089 Milano, Italy; umbertovitale.21@gmail.com

**Keywords:** unicompartmental knee arthroplasty, oxinium, oxidized zirconium, return to sport, patient-reported outcome measures, satisfaction, experience, fixed-bearing, UKA

## Abstract

(1) Background: Unicompartmental knee arthroplasty (UKA) provides a viable alternative to total knee arthroplasty (TKA) in patients with isolated medial osteoarthritis (OA). From 2007 to 2021, 23% of all primary knee arthroplasties in Italy were UKAs. We retrospectively evaluated clinical outcomes and satisfaction in patients implanted with a new oxinium metal-backed fixed-bearing medial unicompartmental prosthesis at a 24-month follow-up. (2) Methods: From December 2020 to December 2021, 145 patients were treated by a single surgeon at a single institution using the hypoallergenic Journey II prosthesis. Clinical outcome measures included the Knee Society Knee Score (KSKS), Knee Society Function Score (KSFS), Oxford Knee Society (OKS) score, University of California Los Angeles Activity Score (UCLA), the Physical Component Summary (PCS), and the Mental Component Summary (MCS), and were calculated preoperatively and at 12 and 24 months. The Forgotten Joint Score-12 (FJS-12) was calculated at 12 and 24 months. Patient satisfaction was collected at 24 months. The scores were compared using the Friedman test. (3) Results: All clinical scores improved significantly from baseline to 24 months (*p* < 0.0001), except for the FJS-12, which from 12 to 24 months did not improve significantly (*p* = 0.041). Patient satisfaction was 9.32 ± 0.74 out of 10. No patient experienced complications or required revision surgery. (4) Conclusions: The Journey II unicompartmental prosthesis is a valuable treatment option for end-stage medial OA, improving knee function, providing pain relief, and ensuring high patient satisfaction at 24 months.

## 1. Introduction

Osteoarthritis (OA) is a degenerative disease where altered joint alignment induces the deterioration of the cartilage weight bearing sheath. With a global prevalence of 16% [1] and an increase of more than 100% since 1990, OA is a major cause of disability worldwide [1,2,3]. The knee is by far the most prevalent site of manifestation, accounting for more than half of OA patients [4]. A single knee compartment is affected in approximately 50% of cases, with the medial compartment being affected five to ten times more frequently than the lateral compartment [5,6].

In advanced knee OA (KOA), joint replacement with an endoprosthesis is a dependable treatment option. According to the Italian Arthroplasty Registry (2022), 215,836 primary knee arthroplasties were performed from 2007 to 2021; of these, 23% (49,627) were unicompartmental knee arthroplasty (UKA) procedures [7].

UKA is a useful option for patients with KOA in the medial or lateral compartment. UKA seems to confer some advantages over total knee arthroplasty (TKA), including a shorter operating time, reduced blood loss, faster recovery, a diminished risk of overall complications, a higher rate of return to activity, and greater cost-effectiveness, while ensuring physiological joint movement [8,9,10,11,12,13]. Furthermore, UKA appears to achieve excellent clinical outcomes more consistently [14,15].

Kozinn and Scott have devised a set of criteria to optimize UKA candidate selection. Although stringent patient selection is key to maximize outcomes, certain aspects of Kozinn and Scott’s original criteria, such as weight, age, activity level, the condition of the patellofemoral joint, and the presence of cartilage calcification, are the subject of ongoing debate [16,17,18].

In recent years, UKA has increasingly been used, particularly to treat medial KOA [19]. Midterm follow-up results indicate survival rates ranging from 90% to 98% at 10 years [20,21,22], while survivorship rates exceeding 90% have been reported in long-term (20 years) studies [23].

In the past decade, minimally invasive surgical techniques for UKA have become increasingly popular [24], even though their highly demanding nature seems to involve suboptimal component alignment, an extended learning curve, and augmented early failure rates [24].

Favorable survivorship outcomes have been documented in UKA implant series [21,25], although registry data indicate higher revision rates compared to TKA procedures [15,26,27]. Several causes can result in the need for UKA revision, including the progression of arthritis in the remaining knee compartments, aseptic loosening [28], and inadequate implant positioning [25,29]. Moreover, the technical intricacies of UKA entail that surgeons with low UKA case volumes may have higher complication rates compared to traditional implant surgery [30].

The increasingly higher number of UKAs performed in the past few years has provided a clearer insight into their complications. Notably, the failures attributed to metal sensitivity have emerged as a significant concern. Allergic reactions in knee arthroplasty have also garnered increased attention. Despite a suboptimal understanding of the immunological mechanisms involved, manufacturers are making substantial investments in and are actively promoting hypoallergenic components, particularly for implantation in individuals sensitive to metals [31,32,33,34,35,36,37,38]. Metal hypersensitivity affects up to 15% of the population [32]. Metal implants have the potential to trigger a type IV hypersensitivity reaction, where macrophage activation, induced by implant debris, leads to the release of interleukin (IL)-1b, the tumor necrosis factor, IL-6, and IL-8. This in turn stimulates osteoclasts, resulting in cutaneous eczematous eruptions, chronic inflammation, pain, and eventually device failure. It may also contribute to osteolysis, metallosis, excessive periprosthetic fibrosis, and muscle necrosis [33,34].

Oxinium (oxidized zirconium; ZrOx) implants have been developed to reduce polyethylene wear and aseptic loosening in knee arthroplasty and have hypoallergenic properties. Besides achieving the objective of improving survival rates [39], their hypoallergenic nature ensures better outcomes in individuals with metal sensitivity [40,41] and in those undergoing revision due to metallosis [42].

ZrOx has been used to manufacture TKA femoral components since the 1980s and total hip arthroplasty (THA) components since 2003 [43]. Heating a zirconium alloy (97.5% zirconium and 2.5% niobium) in the presence of air results in a robust, 5-mm outer oxidized surface that is twice as hard as cobalt–chromium (CoCr) alloy, conferring on the material the strength of a metal while mitigating the risk of the brittle fracture associated with ceramics [43,44,45].

The aims of this study were to determine the survivorship, identify the causes of failure, and assess the functional outcomes of a hypoallergenic, fixed-bearing medial UKA (mUKA) implant, the Journey II (Smith & Nephew, Memphis, TN, USA), at a short-term follow-up. All procedures were performed by a single surgeon at a single institution from 2020 to 2021.

## 2. Materials and Methods

### 2.1. Patient Selection

For this retrospective, single-center cohort study, we mined the database of the Orthopedic Department of Humanitas San Pio X Hospital (Milano, Italy) for all primary mUKA procedures performed with a Journey II implant from December 2020 to December 2023. In total, 581 mUKAs were performed from December 2020 to December 2023; of these, 44 were bilateral. The study complies with the guidelines outlined in the Declaration of Helsinki, as revised in 2013. Following approval by the institutional Review Board, written consent to participate in the study was obtained from all patients.

The data collected from the institutional database included demographic information, the body mass index (BMI), American Society of Anesthesiologists (ASA) class, as well as a medical background, duration of the surgical procedure, duration of hospitalization, any surgical revisions or complications (e.g., intraoperative fractures, postoperative aseptic loosening, infection, stiffness), clinical outcomes, and patient satisfaction data.

### 2.2. Surgical Indications

All mUKA patients had a diagnosis of knee pain and isolated medial unicompartmental KOA with a loss of articular cartilage ≥ grade 3 according to the Kellgren and Lawrence classification [46] or of the spontaneous medial osteonecrosis of the femur with a loss of articular cartilage ≥ grade 3 or minor subchondral collapse. All these patients had already completed a 3-month conservative treatment regimen comprising physical therapy, intra-articular cortisone injections, rest, and anti-inflammatory medications that had, however, failed to reduce their pain symptoms to a tolerable level. Following a comprehensive evaluation of their medical history, a thorough physical examination, and a radiographic assessment involving anteroposterior and anteroposterior weight-bearing X-rays of the full leg as well as lateral weight-bearing knee X-rays including Rosenberg and Merchant views, these individuals were deemed suitable for mUKA [18].

### 2.3. Criteria for Inclusion and Exclusion

Patients were included in the study if they met the classic selection criteria of Kozinn and Scott [47], had undergone mUKA with the Journey II implant, and had a follow-up of at least 2 years. The Kozinn and Scott criteria stipulate a preoperative mechanical axis deformity < 10° in varus or 5° in valgus and a flexion contracture < 15°. The additional criteria were an intact/competent anterior cruciate ligament, an intact lateral compartment, patellofemoral changes not exceeding grade II or III according to the Albach classification [6], and trochlear wear up to grade IV, provided it had a central distribution [48]. The exclusion criteria were primary lateral KOA, a history of complex knee surgery, significant lower limb trauma, fixed varus/valgus deformities, flexion deformities > 15°, inflammatory arthropathy (e.g., rheumatoid arthritis), ataxia, neurological disease, symptomatic KOA in the contralateral knee, and bilateral mUKA or revision surgery (e.g., of a previous mUKA). Additional exclusion criteria were missing data and previous surgery involving the affected knee except arthroscopy for meniscectomy [49]. 

### 2.4. Surgical Procedure

**Figure 1:**
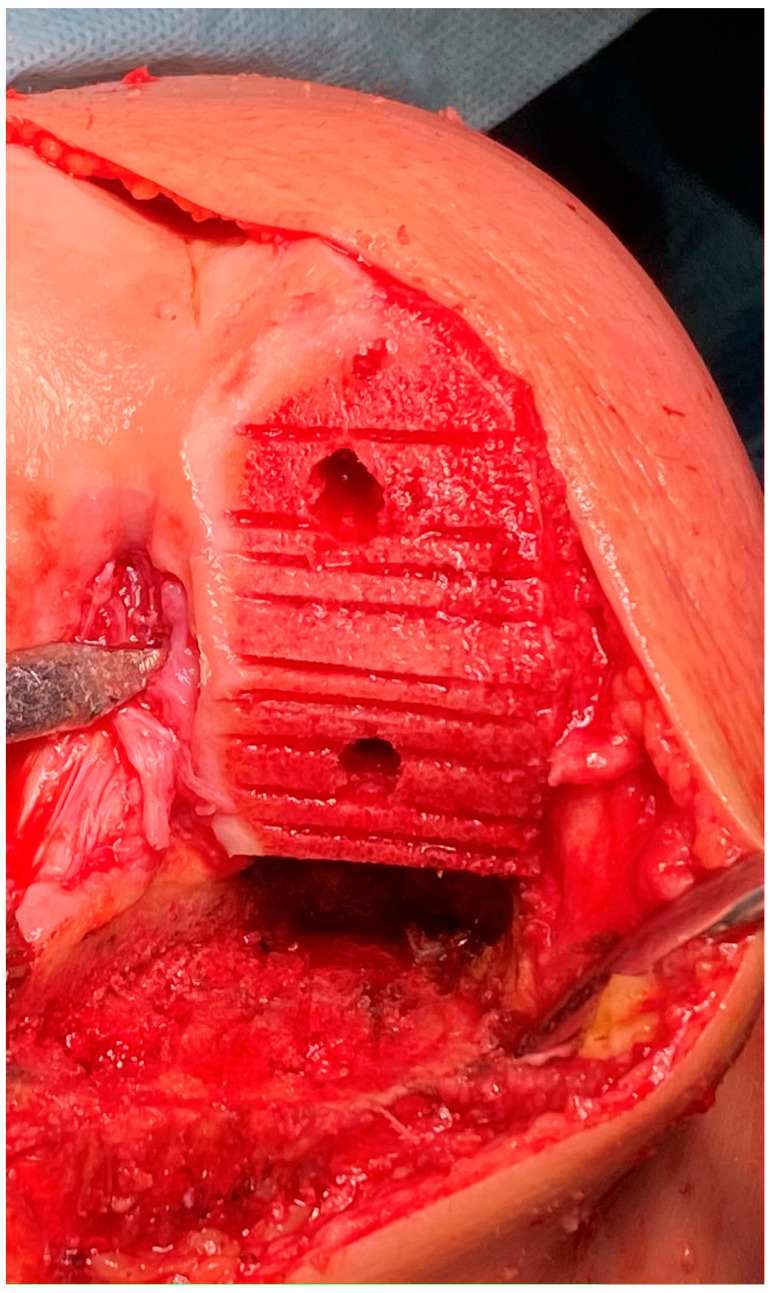
Multiple incisions on medial femoral condyle and medial tibial plateau.

All procedures were performed by the senior surgeon (F.D.A.), who has significant UKA expertise [50]. Prophylactic antibiotics (e.g., cefazolin or vancomycin) were administered perioperatively. The procedures were conducted under spinal anesthesia, complemented by an adductor canal nerve block, without use of a tourniquet or drains. Utilizing an 8–10 cm limited medial midvastus approach, the procedures were executed without inducing lateral patellar subluxation. The surgical steps were in line with the technique recommended by the manufacturer. The tibial coronal cut was executed with due consideration for tibial epiphyseal anatomy. The adjustment of the tibial cutting guide aimed to replicate the angle formed by the tibial joint line and the tibial mechanical axis in the coronal plane, as well as the native tibial slope in the sagittal plane, based on preoperative radiographic measurements. It is usually recommended to aim for a slight undercorrection of the varus limb deformity and to allow for some residual degrees of varus deformity in the coronal alignment of the tibial component [51,52]. Following the removal of the tibial resection, the appropriate trial polyethylene component was positioned, using a trial component of minimal thickness (8 mm) while ensuring adequate flexion and extension gaps. In all cases, the femoral cuts were aligned with the tibial cut surface. To enhance cement penetration, the surfaces were roughened by creating with the saw multiple parallel incisions on the bone perpendicular to the major axis of the condyle, as shown in Figure 1.

Subsequently, a pulse lavage was applied to cleanse the bone surfaces thoroughly. During the procedure, bone cement was applied both to the bony surfaces and to the undersides or backs of the implants. In addition, the fixation pegs on the femoral side were filled with cement. The next step involved the careful removal of excess bone cement and any loose particles, especially in the posterior area of the joint, followed by an inspection of the patellofemoral and lateral compartments.

The primary goals of the procedure were to achieve a balanced flexion-extension gap and restore normal leg alignment. 

### 2.5. Postoperative Care

X-rays were taken and examined within 3 h of the patient leaving the operating room to enable early rehabilitation. Immediate full weight-bearing was initiated. The radiographic follow-up consisted of anteroposterior and lateral weight-bearing knee radiographs taken at 1, 3, 6, and 12 months, and then at yearly intervals. After the operation, all patients received daily prophylaxis with low-molecular-weight heparin for 5 weeks. 

### 2.6. Rehabilitation Protocol

After a review of the postoperative radiographs, patients were allowed full weight-bearing. They were then examined 4 h after the operation and the physical therapist drew up their rehabilitation chart. Active and passive limb mobilization was initiated, with patients wearing elastic stockings on both lower extremities. Patients were then helped to take a short walk using a walker or crutches for support.

### 2.7. Clinical Outcomes

Qualified personnel from the Orthopedic Department collected the key preoperative and postoperative variables before the procedure and then at 12 and 24 months. The major patient-reported outcome measures (PROMs) used were the Knee Society Knee Score (KSKS), the Knee Society Function Score (KSFS) [48], the Oxford Knee Society (OKS) score [53], and the University of California, Los Angeles Activity Score (UCLA) [54]. Further measures, calculated before the procedure and at 12 and 24 months, were the Physical Component Summary (PCS) and the Mental Component Summary (MCS) of the Short Form 36 Health Survey (SF-36), measuring health-related quality of life [55]. The Forgotten Joint Score-12 (FJS-12) was calculated at 12 and 24 months [56]. Patient satisfaction (rated from 1, not satisfied, to 10, completely satisfied) was collected at the last follow-up visit.

### 2.8. Statistical Analysis

All analyses were carried out using Microsoft Excel (version 16.75.2, Redmond, WA, USA) along with the XLSTAT resource pack (XLSTAT-Premium, Addinsoft Inc., New York, NY, USA). The Shapiro–Wilk test was employed to assess whether the data exhibited a non-parametric distribution. Calculated mean values were provided for all continuous data, while percentage frequencies were used for qualitative variables. Baseline and postoperative clinical scores were compared using the non-parametric Friedman test, a method for repeated measures analysis. This test was applied to evaluate differences in the KSKS, KSFS, OKS, UCLA, PCS, and MCS scores between baseline and the next two time points (12 and 24 months). The test was also applied to evaluate the FJS-12 at 12 and 24 months. A *p*-value < 0.05 was considered statistically significant.

## 3. Results

A total number of 145 patients met the study criteria.

### 3.1. Demographics

The participants were 145 patients, 69 men (47.59%) and 76 women (52.41%), who had a mean age of 67.15 years ± 9.38 (range, 49–96). The right knee was affected in 66 patients (45.52) and the left in 79 (54.48%). The mean BMI was 27.63 ± 3.95 kg/m^2^ (range, 19.23–39.26). There were 37 patients (25.52%) with ASA class 1—good physical condition; 90 patients (62.07%) with ASA class 2—slight systemic condition; and 18 patients (12.41%) with ASA class 3—profound systemic condition. The mean operative time was 37.81 ± 6.35 min (range, 20–51). The mean length of hospital stay was 3.32 ± 0.76 days (range, 2–6). These data are reported in Table 1.

### 3.2. Clinical Outcomes

The clinical and functional outcomes of the 145 patients and the results of the statistical analyses are reported in Table 2.

The Friedman test disclosed statistically significant differences in the KSFS, KSKS and OKS UCLA, PCS, and MCS scores between baseline and the two follow-up points (all *p* < 0.0001). The Friedman test, applied to evaluate the FJS-12 scores at 12 and 24 months, failed to highlight any statistically significant differences (*p* = 0.401). Patient satisfaction (rated from 1 to 10) at 24 months was 9.32 ± 0.74 (range, 8–10), as reported in Table 2. 

### 3.3. Complications and Revisions

No patient experienced complications or required revision surgery.

## 4. Discussion

The application of the inclusion and exclusion criteria identified 145 mUKAs with at least a 2-year follow-up. We assessed the clinical and functional outcomes of our patients with the major PROMs, the KSKS, KSFS, OKS, UCLA, PCS, MCS, and FJS-12, and asked them to rate their satisfaction with the results of the procedure using a standard score. All clinical outcome scores except for the FJS-12 reflected excellent results with highly significant differences (*p* < 0.0001) between the preoperative and postoperative follow-up scores. As regards the FJS-12, we compared the values collected at 12 and 24 months (*p* = 0.401). Our data are comparable to those of other mUKA studies and better in some cases [57,58,59,60,61]. The mean satisfaction, rated from 1 to 10, was 9.32 ± 0.74. Several studies have consistently documented satisfaction rates exceeding 90% in mUKA patients [62,63,64]. All our patients returned to sport practice. There were no intraoperative complications like tibial plateau fractures or postoperative complications such as aseptic loosening or infection. We feel this can be attributed to the surgeon’s training and experience and by the relatively short-term follow-up period.

In recent years, the number of mUKA procedures has increased significantly. The reasons for the popularity of mUKA rests on its numerous advantages over TKA, such as a less invasive surgical exposure, the preservation of the native bone stock, retention of the cruciate ligaments, a more limited perioperative morbidity, and expedited postoperative recovery, resulting in greater patient satisfaction. Also, the biomechanics of mUKA implants mimic the native knee function more closely than the TKA implants, thus enhancing dynamic proprioception and postural control [65,66]. In addition, recent investigations have underscored the cost-effectiveness of mUKA when performed in the appropriate patient population [67,68]. 

The value of engaging in low to moderate physical activity is widely recognized, as it contributes to a healthier organism by enhancing physical and social mobility as well as cardiovascular performance. From a public health standpoint, sport practice also reduces healthcare costs, particularly in the age class of the individuals who typically undergo arthroplasty [69]. As knee OA is highly prevalent, it severely affects the ability to practice sports. Some authors have gone as far as to highlight the positive impact of knee arthroplasty on overall health and sports performance [69,70,71]. However, the return to sports after knee arthroplasty is not as extensively studied as other aspects of functional recovery. Our data, specifically the UCLA scores, which reflect a 100% return to sports, agree with the current literature. Notably, Kleeblad and co-workers analyzed UKA patient satisfaction with return to sports and their preferred activities. Compared with the preoperative period, when 81% stated they engaged in sports, 90% were able to do so after the operation. Satisfaction with the return to sports was described by 83%, whereas the return to a higher or similar level was reported in 85.4% of cases [63]. The mean preoperative UCLA score of our patients improved from 5.03 ± 1.61 to 7.48 ± 1.66. In 2018, Lo Presti and colleagues [72] assessed the subjective and objective clinical status of 53 athletic patients subjected to cemented mUKA using the Hospital for Special Surgery (HSS) knee rating score and a visual analog score (VAS). They also evaluated their sporting and recreational activities at a mean follow-up of 48 months. At the last follow-up, 48 of the 53 patients were engaged in sports and recreational disciplines, resulting in a 90% return to activity rate. No early failure and no cases of revision were reported.

The increasingly higher number of UKAs performed in the past few years have provided clearer insight into their complications. Notably, the failures attributed to metal sensitivity have emerged as a significant concern. Allergic reactions in knee arthroplasty have also garnered increased attention. Despite a suboptimal understanding of the immunological mechanisms involved, manufacturers are making substantial investments in and are actively promoting hypoallergenic components, particularly for implantation in individuals sensitive to metals. Metal hypersensitivity affects up to 15% of the population. Metal implants have the potential to trigger a type IV hypersensitivity reaction, where macrophage activation, induced by implant debris, leads to the release of interleukin (IL)-1b, the tumor necrosis factor, IL-6, and IL-8. This in turn stimulates osteoclasts, resulting in cutaneous eczematous eruptions, chronic inflammation, pain, and eventually device failure. It may also contribute to osteolysis, metallosis, excessive periprosthetic fibrosis, and muscle necrosis. In some countries, postoperative complications related to the metal in prosthetic implants, variously defined as metal sensitivity or metal-related pathology, have been found to account for a considerable rate of THA and TKA revisions.

This, to our knowledge, is the first study evaluating the outcomes of the new fixed-bearing Journey II Unicompartmental Knee system, whose femoral component is made of oxinium. 

Whereas allergy to metals is an acknowledged and genuine condition, there is no consensus regarding its clinical implications in routine clinical practice, especially in the context of UKA. A recent study comparing functional outcomes and eosinophil counts in patients with and without a documented history of metal hypersensitivity undergoing UKA has found that 13 out of 128 patients reported a history of metal hypersensitivity prior to the operation. However, the authors found no significant differences in the functional outcomes or eosinophil counts between those 13 patients and the others [36]. Another study has examined whether patients with pain after TKA and metal sensitivity demonstrated improved outcomes after revision with a hypoallergenic implant. Following sensitivity testing, patients underwent revision TKA using either a hypoallergenic component or a standard component. Of the patients who tested positive for metal sensitivity (78.3%), most were sensitive to nickel. Both reactive and non-reactive patients showed significant improvements in their range of motion after revision arthroplasty. The reactive group experienced a significant 37.8% reduction in pain six weeks after revision, whereas the non-reactive group exhibited a moderate, non-significant improvement in pain reduction [37]. In this context, the systemic effects of standard versus hypoallergenic prostheses are a key field of investigation. In 2018, Thomas and colleagues compared two groups of TKA patients at a 5-year follow-up. The only difference between the groups was the type of prosthesis. Half the patients received a standard prosthesis and the other half received a variant characterized by a multilayer advanced surface coating consisting of zirconium nitride (ZrN) to mitigate metal ion release. The implant survival rate (Kaplan–Meier) at 5 years was 97% for uncoated implants and 98% for coated implants. The mechanical axis radiographic outcomes and the Knee Society Score (KSS) pain levels of the two groups were comparable. Although the two groups shared similar values of most serum cytokines, the mean IL-8 and IL-10 levels were higher in the group with the uncoated implant [73]. The coating material commonly used to enable prosthesis implantation in allergic patients is titanium nitride (TiN). Extensive research has demonstrated that TiN can reduce polyethylene wear by as much as 98%. The material is highly resistant to adhesive wear and demonstrates lower adhesion to polyethylene. Importantly, while the CoCr alloy, which is generally used as a coating, catalyzes polyethylene degradation, TiN remains inert. By effectively sealing the CoCr surface, TiN reduces the release of cobalt and chromium ions, thus preventing hypersensitivity reactions [38].

Zirconium, a metal with physical properties similar to titanium, has been employed in knee arthroplasty implants in the form of oxidized zirconium, or “oxinium”. This hybrid material comprises a solid metal core enveloped by a ceramic layer of ZrOx, which is not merely a coating, but forms the surface of the metal alloy. This unique composition combines the surface wear characteristics of ceramic with the internal strength of metal. ZrOx components result in less polyethylene wear compared to CoCr components and exhibit superior resistance to abrasion. An in vitro study has demonstrated a substantial, 42% reduction in polyethylene wear; at 5- and 10-year follow-ups, the survival rate ranged from 95% to 98.7%, with no radiographic failures at either time point [38].

UKA with femoral oxinium has been investigated by D’Ambrosi et al. [12] in a prospective study with a 24-month follow-up. A comparison of UKA in oxinium and in TiNbN (titanium niobium nitride) in patients with metal allergy and isolated anteromedial KOA led the authors to conclude that both helped achieve clinical and radiographic outcomes ranging from good to excellent at the final follow-up, irrespective of age, gender, BMI, bearing type, or implant size. A similar study by Monti et al. [74] described the return to sports of patients implanted with the hypoallergenic prostheses.

Although these studies indicate that oxidized zirconium could extend implant life and reduce the need for revision, there have been reports of catastrophic failure in patients with UKA secondary to the dislocation of fixed polyethylene bearings and metallosis [34,75,76] or with TKA [77,78,79].

Our study has some limitations. Since it was conducted in a high-volume tertiary referral hospital, its findings may not be readily applicable to institutions where UKA is not frequently performed. Further limitations are its retrospective design and the absence of a control group. In addition, given the recent introduction of the Journey II oxinium metal-backed fixed-bearing medial unicompartmental knee system, the follow-up period is necessarily limited to 2 years. It is essential to stress that patient activity levels may change after this relatively short timeframe.

## 5. Conclusions

Our experience with the Journey II mUKA implant showed excellent survival rates at two years, with good to excellent clinical outcomes, a 100% return to sports, and high patient satisfaction. Randomized controlled trials with a longer follow-up period are needed to confirm the present findings with a higher level of evidence. 

## Figures and Tables

**Table 1:** Key data of the 145 medial UKA patients.

Variable	Patients
Age, mean (SD) [range]	67.15 (9.38) [49–96]
Gender	
Male (%)	69 (47.59)
Female (%)	76 (52.41)
BMI (kg/m^2^), mean (SD) [range]	27.63 (3.95) [19.23–39.26]
ASA class (%)	
ASA 1	37 (25.52)
ASA 2	90 (62.07)
ASA 3	18 (12.41)
Operative time (min), mean (SD) [range]	37.81 (6.35) [20–51]
Side	
Right (%)	66 (45.52)
Left (%)	79 (54.48)
Hospital stay (days), mean (SD) [range]	3.32 (0.76) [2–6]

UKA: unicompartmental knee arthroplasty; SD: standard deviation; BMI: body mass index; ASA: American Society of Anesthesiology.

**Table 2:** Preoperative and postoperative clinical and functional data and outcome satisfaction of the 145 medial UKA patients.

Variable	Patients	*p*-Value
KSKS		<0.0001
Preoperative, mean (SD) [range]	44.55 (15.92) [22–70]	
12 months, mean (SD) [range]	87.79 (11.27) [58–97]	
24 months, mean (SD) [range]	89.53 (10.39) [62–100]	
KSFS		<0.0001
Preoperative, mean (SD) [range]	54.83 (15.82) [32–80]	
12 months, mean (SD) [range]	88.44 (10.58) [59–97]	
24 months, mean (SD) [range]	90.99 (10.78) [59–100]	
OKS		<0.0001
Preoperative, mean (SD) [range]	38.57 (7.57) [21–53]	
12 months, mean (SD) [range]	25.01 (5.25) [14–39]	
24 months, mean (SD) [range]	20.29 (5.67) [10–33]	
UCLA		<0.0001
Preoperative, mean (SD) [range]	5.03 (1.61) [1–9]	
12 months, mean (SD) [range]	6.39 (1.47) [3–9]	
24 months, mean (SD) [range]	7.48 (1.66) [3–10]	
PCS		<0.0001
Preoperative, mean (SD) [range]	31.75 (10.73) [6–48]	
12 months, mean (SD) [range]	41.17 (9.58) [13–52]	
24 months, mean (SD) [range]	49.94 (8.41) [26–69]	
MCS		<0.0001
Preoperative, mean (SD) [range]	46.13 (10.71) [15–58]	
12 months, mean (SD) [range]	52.70 (9.65) [30–66]	
24 months, mean (SD) [range]	56.38 (9.54) [33–69]	
FJS-12		0.401
12 months, mean (SD) [range]	83.05 (15.96) [45–100]	
24 months, mean (SD) [range]	85.41 (14.45) [52–99]	
Satisfaction		
24 months, mean (SD) [range]	9.32 (0.74) [8–10]	

KSKS: Knee Society Knee Score; SD: standard deviation; KSFS: Knee Society Function Score; OKS: Oxford. Knee Society; UCLA: University of California, Los Angeles Activity Score; PCS: Physical Component Summary; MCS: Mental Component Summary; FJS-12: Forgotten Joint Score.

## Data Availability

The datasets generated during the current study are available from the corresponding author upon reasonable request.
